# Adenomyosis in patients with rheumatic diseases: a cross-disciplinary clinical observation

**DOI:** 10.3389/frph.2025.1697567

**Published:** 2025-11-18

**Authors:** Silvia Vannuccini, Martina Orlandi, Francesco La Torre, Ernesto Gallucci, Massimiliano Fambrini, Marco Matucci Cerinic, Felice Petraglia

**Affiliations:** 1 Department of Experimental, Clinical and Biomedical Sciences “Mario Serio”, University of Florence, Florence, Italy; 2 Obstetrics and Gynecology, Careggi University Hospital, Florence, Italy; 3 Department of Experimental and Clinical Medicine, University of Florence, Florence, Italy; 4 Rheumatology and Scleroderma Unit, Careggi University Hospital, Florence, Italy

**Keywords:** adenomyosis, dysmenorrhea, heavy menstrual bleeding, pelvic pain, rheumatic diseases, uterine fibroids

## Abstract

Autoimmune and inflammatory rheumatic diseases (RDs) are more prevalent in women and often affect gynecological health. Particularly, heavy menstrual bleeding (HMB) and dysmenorrhea are more common in patients with RD. A link between RDs and endometriosis has been shown, whereas the association with adenomyosis remains unexplored. The present study evaluates the prevalence of adenomyosis in women of reproductive age with RD (*n* = 76) who were referred to the Gynecology Unit, compared with an age-matched control population (*n* = 305). A detailed clinical history and pelvic imaging findings obtained via transvaginal ultrasound were collected, excluding menopausal women and those with endometriosis or gynecological malignancies. Adenomyosis was significantly more prevalent in RD patients than in controls (40.8% vs. 19.7%, OR 2.81, 95% CI 1.64–4.82; *p* < 0.001), whereas the prevalence of uterine fibroids did not differ significantly between groups. These findings highlight the need for greater awareness of adenomyosis among both rheumatologists and gynecologists, as timely and adequate recognition is crucial to improving quality of life and reproductive health in patients with RDs.

## Introduction

Autoimmune and inflammatory rheumatic diseases (RDs) occur significantly more often in women than in men (1, 2), largely due to the influence of sex hormones (3), sex-specific immune regulation (4) and epigenetic mechanisms (1, 5). RDs can significantly affect women's health, and gynecological disorders, such as vaginal dryness, impaired sexuality and infertility, are relevant aspects to consider in patient management (6). In fact, contraception, assisted reproductive technologies, preconception and pregnancy management are topics commonly discussed by RD patients with rheumatologists (7).

Recent studies have shown that women with RDs report menstruation-related symptoms, including heavy menstrual bleeding (HMB), dysmenorrhea and dyspareunia, more often than healthy women did, significantly affecting their quality of life (QoL) (8). These symptoms are common in patients with uterine fibroids—the most common uterine benign tumor (9). However, HMB and gynecological pain are also frequently observed in adenomyosis, a condition characterized by abnormal presence of endometrial tissue within the myometrium (10), which shares several similarities with endometriosis (11), including immune changes (12). The prevalence of adenomyosis varies widely in literature, ranging from 8.8%–61.5%, mainly based on pathological examination of hysterectomy specimens (13). More recently, the use of imaging modalities such as magnetic resonance imaging (MRI) and transvaginal ultrasound (TVUS) has enabled adenomyosis diagnosis in women of reproductive age, with studies estimating a prevalence of approximately 30% in this population (14–16).

While robust evidence on the associations between endometriosis and autoimmune conditions have been reported (17), presumably related to immune dysfunction (18, 19), data on adenomyosis in RD patients remain limited. Thus, the present study aimed to evaluate the possible presence of adenomyosis in a group of RD patients. In addition, the presence of uterine fibroids was systematically investigated.

## Methods

The present observational cohort study included a group of patients with RD, referred from the Rheumatology Unit to the Gynecology Unit at Careggi University Hospital, Florence, over a 1-year period. As part of the multidisciplinary care model implemented at our institution, gynecological consultations are included in the comprehensive evaluation protocol, aiming to either identify potential gynecologic comorbidities or manage gynecologic symptoms related to RDs. A control group of age-matched women (*n* = 305), not affected by RD and undergoing routine gynecological checkups, was also included for comparison.

Our primary objective was to compare the prevalence of adenomyosis in women with RDs vs. age-matched controls. Secondary objectives were to assess the association between adenomyosis and menstruation-related symptoms, including abnormal uterine bleeding (AUB), heavy menstrual bleeding (HMB), dysmenorrhea and dyspareunia; and to compare the prevalence of uterine fibroids between groups.

**Figure 1:**
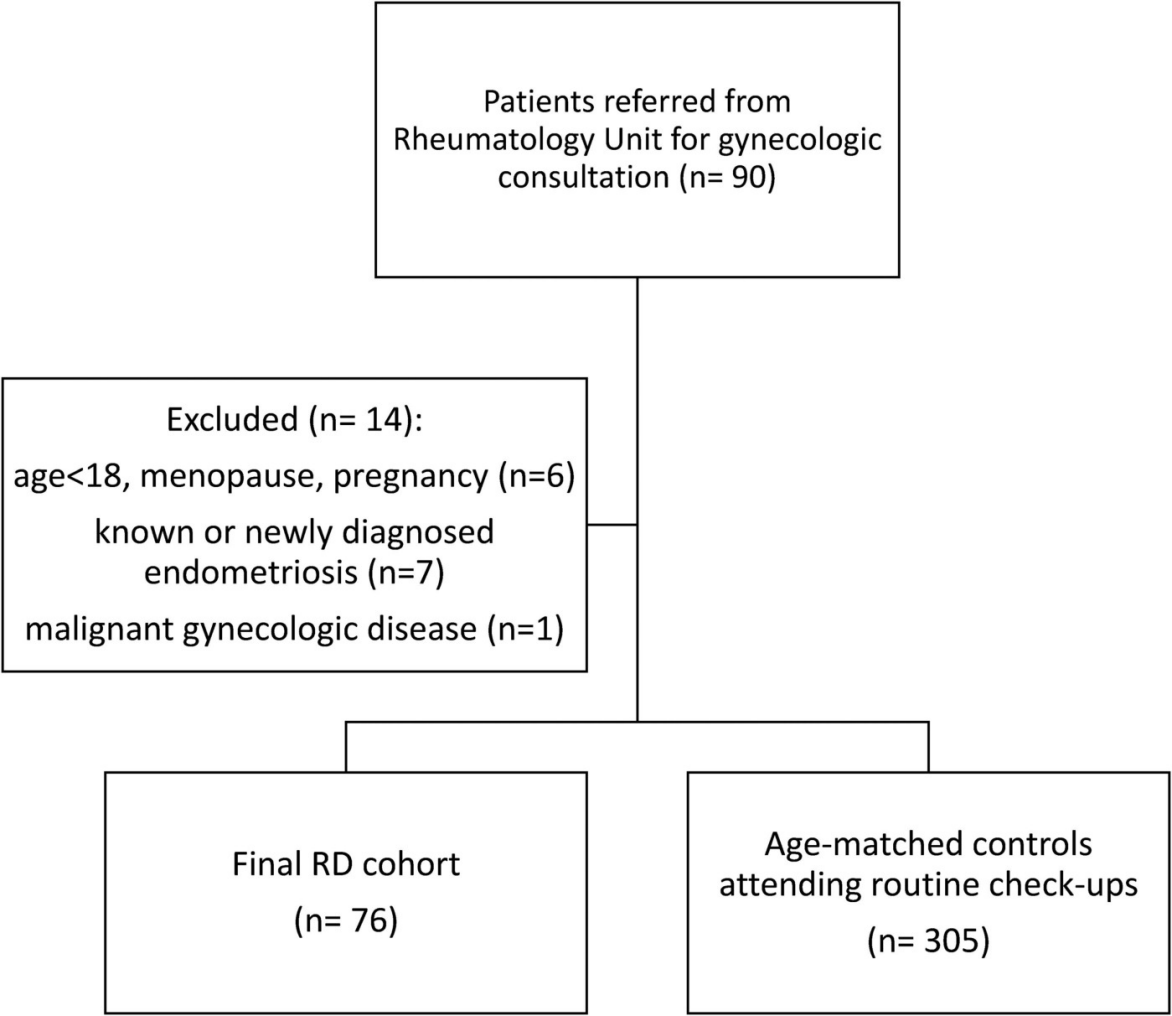
Flow chart of study population, with inclusion and exclusion criteria for eligible participants.

Exclusion criteria included age under 18, overt menopause, pregnancy, and malignant gynecologic diseases. Patients with a known diagnosis of endometriosis or those newly diagnosed with the condition during the gynecological consultation were also excluded from the study. The flow chart outlines the inclusion and exclusion process used to identify eligible participants (Figure 1).

In order to explore the potential diagnosis of adenomyosis, a detailed clinical and gynecological history was obtained from each patient. The presence of AUB, HMB, dysmenorrhea or dyspareunia was assessed. Pain severity was assessed through the Numeric Rating Scale and in the case of 8–10 points, pain was defined as severe. At our institution, ultrasound is routinely performed as part of the standard gynecological consultation. A transvaginal ultrasound (TVUS) pelvic examination was performed by a single operator with expertise in gynecologic imaging (S.V.), using ultrasound machines (Voluson E8, General Electric, GE) equipped with a transvaginal probe (5–7.5 MHz), allowing for two-dimensional (2D), three-dimensional (3D), and Color and Power Doppler evaluation. The diagnosis of adenomyosis was based on a combination of clinical assessment and TVUS findings (10), based the Morphological Uterus Sonographic Assessment (MUSA) consensus. Both direct and indirect MUSA features were considered, including: echogenic subendometrial lines and buds, myometrial cysts, hyperechogenic islands, interrupted or irregular junctional zone, asymmetrical myometrial thickening, globular uterus, translesional vascularity, and fan-shaped shadowing. A diagnosis of adenomyosis was made when at least one direct feature was identified (14, 20). In addition to the assessment for adenomyosis, the presence of uterine fibroids was also evaluated (21, 22). Suspected endometriosis was systematically screened and excluded according to the International Deep Endometriosis Analysis (IDEA) consensus protocol (23).

The study was approved by the local Ethics Committee (protocol number 14558). Informed consent was obtained from the subjects involved in the study.

All the data were reported in an electronic database and the statistical analysis was performed by using IBM SPSS Statistics software, version 22 (IBM Corporation, Armonk, NY, United States). Descriptive statistics were performed: continuous variables are expressed as the mean (±standard deviation, SD), whereas for categorical data, absolute and relative frequencies were calculated. Normality of continuous variables was assessed with the Shapiro–Wilk test. Continuous parameters were evaluated by Student's t test for normally distributed data, otherwise the Mann–Whitney test was applied. To evaluate the associations between categorical variables and patient groups a chi-square test or Fisher's exact test were used. Effect sizes for categorical outcomes were expressed as odds ratios (OR) with 95% confidence intervals, derived from 2 × 2 contingency tables. Additionally, a multivariable logistic regression analysis was performed to estimate the association between RD status and adenomyosis, adjusting for potential confounders. Variables included in the model were age, BMI, nulliparity, previous caesarean section, and current hormonal therapy. These covariates were selected *a priori* based on clinical relevance and data availability. Results are reported as adjusted odds ratios (aOR) with 95% confidence intervals (CI). A *p*-value < 0.05 was considered statistically significant.

## Results

The study population included 76 patients with RD (mean age 39 ± 8.48 years). RDs were distributed as follows:

1. connective tissue diseases (52%): Sjögren's syndrome, systemic lupus erythematosus, systemic sclerosis, and undifferentiated connective tissue disease;
2. arthritis (41%): rheumatoid arthritis, spondyloarthritis, juvenile idiopathic arthritis, polyarthritis, psoriatic arthritis, other seronegative spondylarthritis;
3. vasculitis (7%): Behçet's disease;

Almost all RD patients (98.6%) were receiving treatment for their underlying disease. Baseline demographic and clinical characteristics of RD patients and controls are summarized in Table 1. No significant differences were observed between the two groups in term of age, nulliparity, infertility, previous caesarean section and current hormonal treatment. However, when evaluating menstrual cycle symptoms and gynecological pain, women with RDs reported significantly higher rates of AUB (39.5% vs. 26.2%; *p* = 0.025), HMB (43.4% vs. 25.9%; *p* = 0.004) and severe dysmenorrhea (55.2% vs. 21.3%; *p* < 0.001) compared to controls (Table 1).

**Table 1:** Gynecological history and symptoms of RDs patients and controls.

Variables	RDs patients *n* = 76	Controls *n* = 305	*p*
Age	39 ± 8.5	39 ± 8.0	0.970
BMI	23.3 ± 6.5	22.9 ± 5.5	0.4578
Menarche	12.7 ± 1.7	12.4 ± 1.6	0.208
Nulliparity	40 (52.6%)	143 (46.8%)	0.373
Recurrent pregnancy loss	4 (5.2%)	19 (6.2%)	1.000*
Infertility	11 (14.4%)	25 (8.1%)	0.122*
Previous CS	16 (20.8%)	56 (18.5%)	0.624
Current hormonal therapy	10 (13.2%)	61 (20.0%)	0.18*
Menstrual cycle			
Irregular in frequency	16 (21%)	61 (20%)	0.873
AUB	23 (30.2%)	55 (18%)	0.025
HMB	33 (43.4%)	79 (25.9%)	0.004
Dysmenorrhea	56 (73.6%)	120 (39.3%)	<0.001
Severe dysmenorrhea	42 (55.2%)	65 (21.3%)	<0.001
Dyspareunia	11 (14.4%)	25 (8.1%)	0.122

Data are presented as *n* (%) for categorical variables and as mean ± SD or median (IQR) for continuous variables according to distribution (Shapiro–Wilk). *P*-values were calculated using Student's *t*-test for normally distributed continuous variables, Mann–Whitney *U* test for non-normally distributed continuous variables, and chi-square test or Fisher's exact test (*) for categorical variables, as appropriate.

In RD patients, current hormonal therapy consisted of progestin-only regimens (oral progestins or levonorgestrel-releasing intrauterine device). In controls, hormonal therapy also included combined oral contraceptives.

BMI, body mass index; CS, caesarean section; AUB, abnormal uterine bleeding; HMB, heavy menstrual bleeding; Severe dysmenorrhea was defined as NRS 8–10, NRS, numeric rating scale.

**Figure 2:**
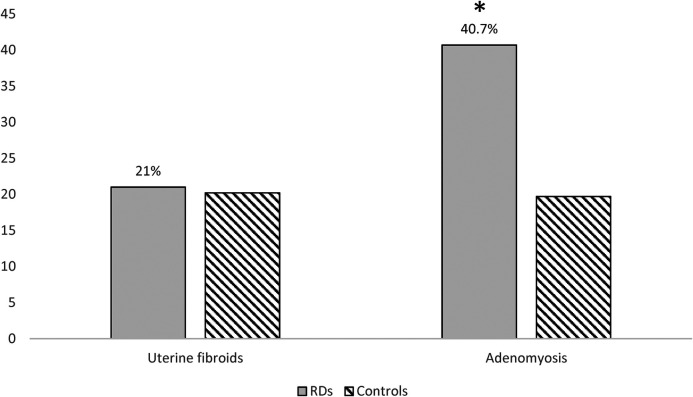
Adenomyosis and uterine fibroids in RDs and controls. *p value < 0.05.

Among the 76 women with RDs and the 305 age-matched controls included, the prevalence of adenomyosis was significantly higher in RD patients (31/76, 40.8% vs. 60/305, 19.7%; OR 2.81, 95% CI 1.64–4.82; *p* < 0.001) (Figure 2). Table 3 summarizes the frequency of various RDs within the study population and the related prevalence of adenomyosis observed across these groups. After adjustment for age, BMI, nulliparity, previous caesarean section, and current hormonal therapy, RD remained independently associated with adenomyosis (aOR 2.60, 95% CI 1.44–4.71; *p* = 0.002). None of the covariates showed significant independent associations (Table 2). Adenomyosis was also significantly associated with the clinical coexistence of HMB and severe dysmenorrhea (OR = 5.75 (CI 95%: 2.08–15.88; *p* = 0.0009).

**Table 2:** Multivariable logistic regression for adenomyosis.

Variable	aOR	95% CI	*p*
RD	2.60	1.44–4.71	0.002
Age	1.01	0.98–1.04	0.50
BMI	1.02	0.98–1.06	0.33
Nulliparity	1.20	0.62–2.34	0.61
Previous CS	1.10	0.59–2.05	0.74
Current hormonal treatment	0.82	0.41–1.64	0.35

aOR, adjusted odds ratio; CI, confidence interval; CS, caesarean section.

**Table 3:** Prevalence of adenomyosis across RD subtypes.

RD subtype	RD patients	Adenomyosis prevalence
Sjögren's syndrome	22 (29.1)	13 (59.1)
Rheumatoid arthritis	10 (12.7)	4 (40.0)
Lichen	10 (12.7)	3 (30.0)
Behçet's disease	7 (9.1)	3 (42.9)
Systemic lupus erythematosus	1 (1.8)	0
Raynaud's syndrome	1 (1.8)	0
Ankylosing spondylitis	6 (7.3)	2 (33.3)
Systemic sclerosis	1 (1.8)	0
Juvenile idiopathic arthritis	6 (7.3)	2 (33.3)
Polyarthritis	1 (1.0)	0
Undifferentiated connective tissue	4 (5.5)	2 (50)
Psoriatic arthritis	6 (7.3)	2 (33.3)
Other seronegative spondyloarthritis	1 (1.8)	0
Total	76	31 (40.8)

Data are presented as *n* (%). Values refer to the number of patients in each RD subtype and the corresponding prevalence of adenomyosis.

In contrast, the prevalence of uterine fibroids did not differ between groups (16/76, 21.1% vs. 61/305, 20.2%; *p* = 0.873), with the coexistence of both conditions in 15.3% cases of RD patients (Figure 2).

## Discussion

The primary finding of our study is a significantly higher prevalence of adenomyosis in women with RDs compared to age-matched controls. This association remained significant after multivariable adjustment for age, BMI, nulliparity, previous caesarean section, and current hormonal therapy. This novel association suggests a link between immune-mediated rheumatic conditions and adenomyosis, in line with existing evidence on endometriosis and autoimmune disorders (17). Furthermore, patients with RDs were overall significantly more symptomatic, experiencing higher frequencies of AUB, HMB and severe dysmenorrhea than the control group. Notably, the coexistence of adenomyosis and RDs was significantly associated with both HMB and severe dysmenorrhea.

The interplay between sex hormones and immunity appears to be a central mechanism to explain this pattern. Estrogen and progesterone modulate immune responses by influencing lymphocyte activity, cytokine production, and antibody synthesis. In women with RDs—who already exhibit immune dysregulation—this hormonal–immune interaction could exacerbate menstrual symptoms via heightened inflammatory responses in the endometrium (24). These symptoms, including AUB, HMB, and dysmenorrhea, are also key clinical features of structural uterine disorders such as adenomyosis (10, 16). Nevertheless, bleeding symptoms referred by patients affected by either uterine fibroids or adenomyosis are strongly associated with reduced quality of life and increased perceived stress (25). In our cohort, adenomyosis was significantly more frequent in women with RDs and was itself significantly associated with the coexistence of HMB and severe dysmenorrhea, suggesting a compounding effect of systemic inflammation and uterine pathology.

The pathogenetic mechanism linking adenomyosis and RD may involve inflammatory and immune dysregulation (26, 27), as it is reported in endometriosis (28). In adenomyosis, both systemic and local immune changes, through the epithelial-mesenchymal transition mechanism, stimulate the migration of endometrial cells into the myometrium and promote inflammation (12). Adenomyosis is also characterized by hormonal aberrations, such as increased estrogen receptors activity and progesterone resistance (29, 30). Estrogen and progesterone modulate the immune system, affecting the cascade of proinflammatory factors (31–33). Immune dysregulation and inflammation may contribute to menstruation-related disorders in women with RDs (12, 17, 34–36). The RD inflammatory setting may amplify the local inflammation characteristic of adenomyosis and potentially exacerbate menstrual symptoms (37). In RDs neurogenic inflammation may also play a role in amplifying gynecological pain symptoms. By altering neuronal excitability and impairing pain sensitivity, it contributes to heightened pain perception and central sensitization, thereby potentially worsening dysmenorrhea and other pelvic pain manifestations (38). Importantly, almost all RD patients in our cohort were receiving treatment for their underlying disease. However, we did not investigate whether rheumatologic therapy influenced adenomyosis-related symptoms, nor whether adenomyosis management could impact the course of RD. Furthermore, we did not collect data on disease duration or severity, which may also play a role in the association between RDs and adenomyosis. Regarding hormonal therapy, while its overall prevalence did not significantly differ between groups, RD patients were treated exclusively with progestin-only regimens, whereas combined oral contraceptives were also represented among controls. This heterogeneity may have influenced menstrual symptom profiles, despite the relatively small proportion of patients on hormonal treatment in both groups. These important questions remain unexplored in the current literature and warrant further dedicated studies.

Interestingly, the study found a significantly higher incidence of adenomyosis in women with RDs, independent of endometriosis as a comorbidity. It is already well established that RDs are associated with an increased prevalence of endometriosis (17), which is frequently underdiagnosed and may occur alone or in combination with adenomyosis. Indeed, endometriosis shares several pathophysiological and immunological features with adenomyosis (11). To minimize confounding, women with a known or newly diagnosed endometriosis were excluded, allowing a clearer exploration of the relationship between adenomyosis and RDs.

Regarding uterine fibroids, no significant difference in prevalence was observed between women with RDs and controls groups, supporting the concept that fibroids may represent a distinct clinical and biological entity compared to adenomyosis, despite partially overlapping clinical manifestations, including AUB, HMB, and pelvic pain (39). The pathogenesis of fibroids is multifactorial, involving hormonal, genetic, and environmental factors, but growing evidence indicates that immune and inflammatory processes also contribute to their development and progression. Fibroids are associated with chronic, low-grade inflammation in the myometrium, characterized by infiltration of immune cells such as macrophages, mast cells, and T lymphocytes. These cells release mediators that promote fibrosis, angiogenesis, and tissue growth, while impaired immune tolerance and abnormal responses to injury may also contribute. However, unlike adenomyosis, where inflammation is central to disease mechanisms, in fibroids these pathways are secondary, with pathogenesis driven primarily by hormonal signalling and somatic mutations in smooth muscle cells (39). For this reason, the lack of a higher fibroid prevalence in RD patients suggests that systemic autoimmune and inflammatory processes, which strongly intersect with adenomyosis, may not significantly influence fibroid development. Assessing both conditions in the same cohort is nonetheless crucial, as it allows for differentiation between overlapping gynecologic symptoms and provides a clearer understanding of the specific uterine pathologies more closely linked to RDs.

Some limitations of this study should be acknowledged. First, no *a priori* sample-size calculation was performed, as this was an exploratory study analyzing a consecutive cohort within a multidisciplinary care pathway. Despite that, the observed difference in adenomyosis prevalence between groups was large and statistically robust, suggesting that the available sample size was adequate to detect clinically relevant associations. Nevertheless, the overall number of RD patients was relatively limited, and RDs are highly heterogeneous in their clinical manifestations. The study was therefore not powered to perform subgroup analyses across individual RD subtypes. Moreover, the single-centre design may limit generalizability. Although no specific distribution of uterine disorders was observed across different RDs, the variability within this patient population may be an additional limitation. The potential for selection bias should also be considered, as participation in the gynecological consultation was voluntary. Additionally, the operator was not blinded to RD status, since the examinations were conducted in a real-world multidisciplinary setting. Although the consultation was systematically offered to all women with RDs within a multidisciplinary care setting, it is possible that those experiencing gynecological symptoms were more likely to accept referral, potentially leading to an overrepresentation of symptomatic patients in the study cohort. Age distribution also represents a relevant consideration. All participants were over 30 years of age, reflecting both the typical age at diagnosis of most RDs and the deliberate exclusion of patients with endometriosis. Since endometriosis is often diagnosed earlier in reproductive life, its exclusion may have skewed the population toward older age groups, in whom adenomyosis is more prevalent. As a result, the findings may not fully extend to younger RD patients. Nonetheless, as endometriosis can sometimes be asymptomatic or undetectable on imaging, its complete exclusion cannot be assured. This limitation was partly mitigated by the use of the IDEA protocol, which maximized diagnostic accuracy and reduced, though did not entirely eliminate, the risk of missed or subclinical cases.

Despite these limitations, the study has several notable strengths. To our knowledge, this is the first report to systematically evaluate menstruation-related disorders other than endometriosis in RD patients, providing novel insights into a relatively unexplored area of women's health in rheumatology. In addition, the inclusion of an age-matched control group represents a methodological strength, as both adenomyosis and uterine fibroids increase with age. Matching minimized the potential confounding effect of age and strengthened the reliability of comparisons between RD patients and controls. Finally, all ultrasound assessments were performed by an experienced sonographer using strict protocols (23, 25) ensuring high diagnostic accuracy and consistency across the study.

In conclusion, this study highlights a significantly higher prevalence of adenomyosis in women with RDs, with important implications for both clinical practice and future research. These findings suggest a potential role of systemic inflammation and immune dysregulation in adenomyosis pathophysiology and underscore the value of interdisciplinary management. Greater awareness of this association is crucial for rheumatologists and gynecologists, as early recognition of menstrual symptoms such as HMB or severe dysmenorrhea should prompt timely gynecologic referral, given that RD patients are at increased risk for adenomyosis. Considering the well-documented impact of adenomyosis on fertility and pregnancy outcomes (40), its coexistence with RDs may further exacerbate reproductive risks already present in this population (41, 42). Strengthening collaboration between rheumatology and gynecology, alongside continued investigation into shared mechanisms, may lead to earlier diagnosis, improved patient care, and the development of targeted therapeutic strategies.

## Data Availability

The raw data supporting the conclusions of this article will be made available by the authors, without undue reservation.
